# Epidural Blood Patch Using a Racz Catheter for Spontaneous Intracranial Hypotension With Unclear Leak Points

**DOI:** 10.7759/cureus.23559

**Published:** 2022-03-28

**Authors:** Megumi Kanao-Kanda, Satoru Hiroshima, Izumi Sato, Ririko Nagabuchi, Hirotsugu Kanda

**Affiliations:** 1 Anesthesiology and Critical Care Medicine, Asahikawa Medical University, Asahikawa, JPN; 2 Neurosurgery, Asahikawa Medical University, Asahikawa, JPN

**Keywords:** spontaneous intracranial hypotension, orthostatic headache, cerebrospinal fluid leakage, epidural blood patch, racz catheter

## Abstract

Using a Racz catheter (Brevi-XL™, Epimed Inc., NY, USA) to insert an epidural blood patch (EBP) may be an effective method of reaching the target epidural space in the cervical region. We would like to present a case, wherein a targeted EBP via Racz catheter was used in the management of spontaneous intracranial hypotension. When the leak point is clear via imaging, EBP should be performed exactly at that point. However, if the leak point is unclear, with only a contrast agent pool detected via imaging, EBP should be performed to mask the entire region of the pool. In both cases, EBP via Racz catheter is a convenient and effective method for the management of spontaneous intracranial hypotension. Further cases may be needed to verify our results.

## Introduction

Due to the leakage of cerebrospinal fluid (CSF), spontaneous intracranial hypotension (SIH) causes headache, dizziness, and nausea. Diagnosis of SIH is made by contrast-enhanced brain magnetic resonance imaging (MRI), radionuclide angiography, and computed tomography (CT) myelography. SIH is often treated with an epidural blood patch (EBP) [1]. We performed EBP using Racz catheters (Brevi-XL™, Epimed Inc., NY, USA). It helps to target the leak because the tip of the catheter can easily reach the leak site under fluoroscopy guide. Racz catheters have been manufactured specifically as epidural catheters for epidural anesthesia, but their successful use in EBP has only recently been reported [2,3]. Performing an EBP using a Racz catheter may be an effective way to reach the target epidural space in the neck. In this report, we present a case in which targeted EBP via a Racz catheter was effective in managing SIH even when the leak point was unclear.

## Case presentation

**Figure 1:**
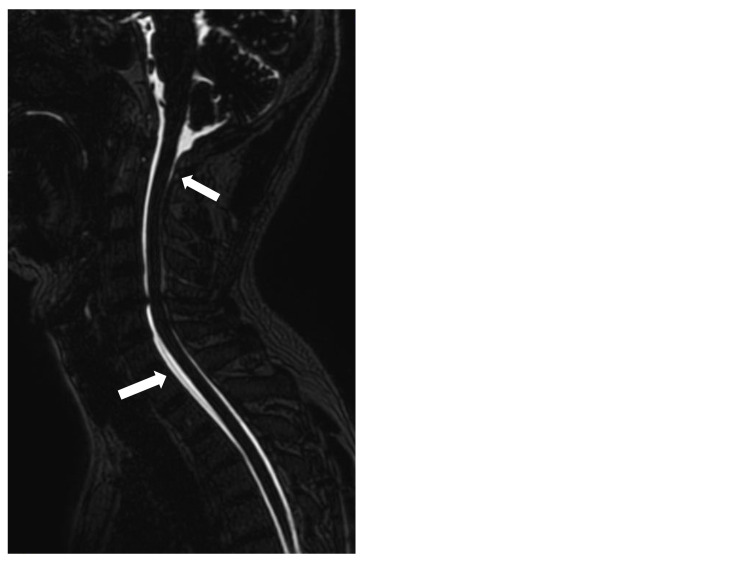
Cervical MRI image showed CSF leakage around the C1-2 and C7-T2 levels. White arrows indicate CSF leak around C1-2 and C7-T2 levels.

A 46-year-old male patient with no history of trauma developed a worsening traction headache, which hindered his ability to perform manual labor work. He was diagnosed with SIH because he had the following symptoms: a typical orthostatic headache that worsened when he stood up and disappeared when he lay down, and CSF leakage according to his cervical MRI. The cervical MRI scan revealed a pool of CSF leakage around the C1-2 and C7-T2 levels with no clear leak point (Figure 1). Written informed consent was obtained from the patient for publishing this case.

CT myelography also showed CSF pool in the cervical epidural space, but no leak points were detected. After consulting with neurosurgery, we decided to perform EBP using a Racz catheter for treatment. The patient consented to the EBP procedure after the risks and benefits were thoroughly explained.

**Figure 2:**
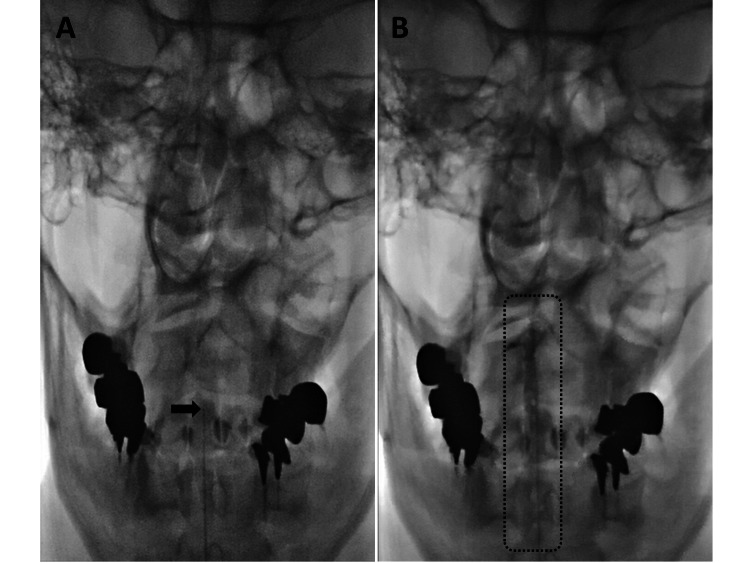
X-ray image. X-ray image of the front before (A) and after injection of blood and contrast agent (B). The black arrow indicates the tip of the Racz catheter (A). The dotted-line rectangle indicates the contrast agent (B).

A fluoroscopy-guided EBP via Racz catheter with a liquid mixture of autologous blood and a contrast agent was performed [2]. In the operating room, the patient was placed in the prone position with a high pillow under his chest to open the interspinal space. In this position, a 15-gauge epidural needle was inserted at the T4-5 interlaminar level using a paramedian approach, and then under fluoroscopy guidance, the needle was advanced by the loss-of-resistance technique. After the needle reached the epidural space, the Racz catheter was advanced toward the cephalad direction. The tip of the catheter reached C1-2 level under fluoroscopic guidance without any difficulty. The cervical epidural space was confirmed via a contrast agent (Figures 2A, 2B).

**Figure 3:**
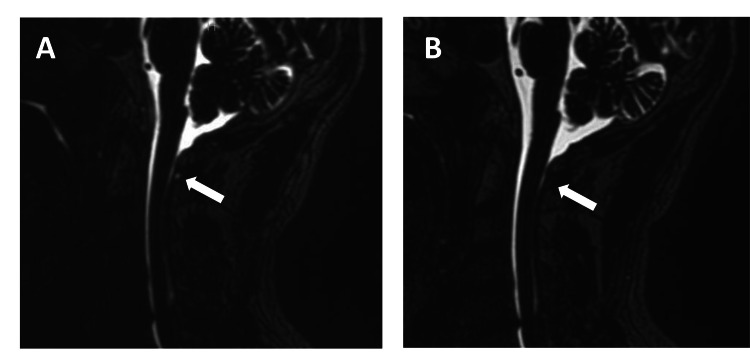
Cervical MRI image. Cervical MRI image of before (A) and after EBP (B). White arrow indicates CSF leak around C1-2 (A). The CSF leakage is not observed after EBP (white arrow) at the same point (B). EBP: epidural blood patch.

Subsequently, a total of 30 mL of the mixture containing 24 mL of autologous blood and 6 mL of contrast agent was injected into the epidural space through the catheter. The mixture was administered in two places: 10 mL and 20 mL were injected into C1-3 and C6-T3, respectively. The patient’s orthostatic headache immediately disappeared. He was discharged four days after his treatment without any complications. The CSF leakage pool that was observed on MRI before EBP disappeared after EBP (Figures 3A, 3B).

 The patient could engage in manual labor, without symptoms, for more than 18 months.

## Discussion

The first-line treatment for SIH is conservative therapies such as hydration, dosing, and abdominal binders [1]. EBP is performed when the conservative management described is not effective. Traditionally, due to anatomical complexity, blind lumbar EBP has sometimes been selected, even for cervical leaks. However, EBP success rate for SIH ranges from about 50% to 65%, an inadequate effect in clinical practice [4]. In contrast, Inamasu and Nakatsukasa have reported a targeted EBP procedure for C1-2 leaks using an epidural catheter from the lower cervical spine [5].

Recently, our study and the study by Kim reported cases of SIH treated with targeted cervical EBP using a Racz catheter [2,3]. Traditional EBP, especially cervical EBP, has a potential risk of causing spinal or radiculopathy damage due to anatomical complexity. On the other hand, EBP via a Racz catheter is a useful treatment because it can be performed without puncturing a needle into an anatomically complex area such as cervical epidural space. The tip of the Racz catheter is soft, elastic, and relatively sturdy to prevent twisting and provide excellent handling. The Racz catheter is a radio-opaque spiral stainless steel coil. An advantage of the Racz catheter is that setting the catheter tip at the fluoroscopically guided cervical leakage point is easier than that at the conventional epidural catheters. Conversely, there may be disadvantages to using a Racz catheter for EBP. Due to the small lumen of the Racz catheter, the catheter may become stuffed during EBP due to blood clotting [6].

In our previous case, the Racz catheter tip targeted the obvious leak site (C2) when inserted through C7-Th1 [2]. It was confirmed that blood and contrast media were used to visualize the diffusion and EBP was performed to ensure closure of the cervical leak site. Unlike the previous case report presented by us and Kim, in this case, the leak point was unclear. In patients with SIH, CT myelography is a more accurate indicator of CSF leakage than other imaging modalities [7]. However, Hashizume et al. also reported that CSF leakage may not be detected by CT myelography with radioisotope cisternography in patients with traumatic CSF leakage [8].

Hatano et al. reported a case of SIH treated with EBP via a Racz catheter from top to bottom of the epidural space, that is, from the neck to the lumbar region [6]; they show that single-entry multisite EBP is effective when the area of CSF leakage is large and suggest that EBP via a Racz catheter may be useful when the site of CSF leakage is unclear. We agree with their assumption from the experience of this case.

When the leak point is clear via imaging, EBP should be performed exactly at that point. However, if the leak point is unclear, with only a contrast agent pool detected via imaging, EBP should be performed to mask the entire region of the pool. In both cases, EBP via a Racz catheter is an effective method. EBP via a Racz catheter, which allows extensive treatment with a single needle puncture, is also safer, especially if there are many or widespread areas of potential CSF leakage.

## Conclusions

Once the leak point is clarified by imaging, EBP needs to be performed exactly at that point. However, in this case report, where the leak point was unclear and the imaging only detected the contrast agent pool, EBP was performed to mask the entire area of the pool. In both cases, EBP via the Racz catheter is a convenient and effective method for managing SIH. Further case studies may need to be conducted to verify our results.
